# Human Skin Bacterial Community Response to Probiotic (*Lactobacillus reuteri* DSM 17938) Introduction

**DOI:** 10.3390/microorganisms8081223

**Published:** 2020-08-11

**Authors:** Marie Frerejacques, Camille Rousselle, Loüen Gauthier, Salomé Cottet-Emard, Léa Derobert, Anne Roynette, Thomas Z. Lerch, Frédérique Changey

**Affiliations:** 1 EBInnov, School of Industrial Biology—EBI, 49 Avenue des Genottes, 95800 Cergy, France; m.frerejacques@hubebi.com (M.F.); c.rousselle@hubebi.com (C.R.); l.gauthier@hubebi.com (L.G.); s.cottetemard@hubebi.com (S.C.-E.); l.derobert@hubebi.com (L.D.); a.roynette@hubebi.com (A.R.); 2 IEES-Paris, UMR 7618 (Sorbonne Université, IRD, CNRS, INRA, UPEC, Université de Paris), 61 Avenue du Général de Gaulle, 94010 Créteil, France; thomas.lerch@u-pec.fr

**Keywords:** skin microbiota, *Lactobacillus reuteri* DSM 17938, bacterial introduction, T-RFLP, resilience

## Abstract

The introduction of a strain or consortium has often been considered as a potential solution to restore microbial ecosystems. Extensive research on the skin microbiota has led to the development of probiotic products (with live bacterial strains) that are likely to treat dysbiosis. However, the effects of such introductions on the indigenous microbiota have not yet been investigated. Here, through a daily application of *Lactobacillus reuteri* DSM 17938 on volunteers’ forearm skin, we studied in vivo the impact of a probiotic on the indigenous skin bacterial community diversity using Terminal-Restriction Fragment Length Polymorphism (T-RFLP) for 3 weeks. The results demonstrate that *Lactobacillus reuteri* DSM 17938 inoculum had a transient effect on the indigenous community, as the resilience phenomenon was observed within the skin microbiota. Moreover, *Lactobacillus reuteri* DSM 17938 monitoring showed that, despite a high level of detection after 2 weeks of application, thereafter the colonization rate drops drastically. The probiotic colonization rate was correlated significantly to the effect on the indigenous microbial community structure. These preliminary results suggest that the success of probiotic use and the potential health benefits resides in the interactions with the human microbiota.

## 1. Introduction

The human skin constitutes a protective barrier to prevent external aggressions and dehydration [1]. On average, this organ exceeds two square meters [2] and accounts for approximately 16% of the human body weight [3]. It is also a habitat for numerous microorganisms, such as bacteria, archaea, fungi, viruses, and acarids [4], which perform a wide range of microbial functions. The human skin microbiota gets enriched during the course of a lifetime and includes between 400 and 1000 bacterial species that can reach a total abundance of 1 million per square centimeter [5]. The interpersonal variability of the cutaneous microbiota structure depends on several factors: lifestyle, hygiene and food, target population, gender, age, and ethnicity or genetic background [6,7,8,9]. These factors, as well as environmental ones, could be responsible for skin physiological dysbiosis, which leads to moderate or severe diseases [10]. Several studies have been undertaken to unlock the skin microbial black box and to understand the relationship between the microbiota and skin physiology. The use of bacterial inoculum (referred to as probiotics) is one of the alternative treatments for skin disease. However, the skin is not only an organ but also a complex microbial ecosystem. Defined as such, the ecological balance rests on interactions between microorganisms and their natural biotic and abiotic environments [11]. Thus, the success of the probiotic introduction must be evaluated not only on its ability to colonize the skin habitat but also on the potential positive or negative effects generated on the indigenous microbial network. The application of microbial ecological concepts offers a useful framework for improving the understanding of the relationship of the human microbiome with human health [12].

According to definitions by the Food and Agriculture Organization of the United Nations and the World Health Organization, a probiotic consists of live microorganisms which, when administered in sufficient amounts, confer a health benefit on the host [13]. The effect of probiotics resides more in their ability to share beneficial genes and metabolites with indigenous microbiota than in their ability to colonize [14]. Studies dealing with the effect of probiotics on the structure of indigenous microbial communities from different human microbiota have often yielded contradictory results, with no effect [15], short-term effects [16], or significant long-term effects [17]. In regard to the skin, a recent study showed that the human skin microbiota can be transplanted from a donor to a recipient on a cleaned and disinfected area [18]. To our knowledge, the effect of probiotic introduction on the indigenous skin microbiota has never been studied yet.

The objective of this small and preliminary investigation is to evaluate the impact of a modified *Lactobacillus reuteri* strain introduction on human skin bacterial communities. *Lactobacillus reuteri* species are Gram-positive lactic acid bacteria present in the commensal microbiota of many animals and identified as potential probiotics [19,20]. *Lactobacillus reuteri* DSM 17938 (*L. reuteri* DSM 17938) is a commonly used probiotic strain modified from *Lactobacillus reuteri* ATCC 55730, obtained by the removal of the two plasmids to avoid antibiotic resistance dissemination in the environment without a change in the probiotic properties. *Lactobacillus reuteri* DSM 17938 presents the same fermentation pattern and the ability to produce reuterin [21]. Reuterin is a powerful broad-spectrum antimicrobial substance with a low molecular weight and that is a neutral, water-soluble compound. It has been shown that reuterin can inhibit the growth of bacteria belonging to various genera, such as *Escherichia*, *Salmonella*, *Shigella*, *Proteus*, *Pseudomonas*, *Clostridium*, and *Staphylococcus* [22]. Currently, *L. reuteri* DSM 17938 is commercialized as a probiotic for the gut, especially in the management of infant colic. Recent in vitro experiments on Reconstructed Human Epidermis (RHE) have demonstrated that the spreading of *L. reuteri* DSM 17938 cells has a positive impact on inflammatory skin disorders [23]. In the present short study, we conducted an experiment on a panel of healthy young women. During 3 weeks of daily introduction of *L. reuteri* DSM 17938 on the skin, we followed the colonization of the probiotic and the structure of the indigenous bacterial community using a molecular fingerprint method. We hypothesized that *L. reuteri* DSM 17938 colonization and its effects on indigenous microbiota would be weak or transitory. Although the experimental panel was considered as homogenous, we also expected that the variability in skin microbial communities between individuals would also influence both probiotic colonization and the response of the indigenous microbiota.

## 2. Materials and Methods

### 2.1. Inoculum Conception

*Lactobacillus reuteri* DSM 17938 was obtained by Biogaia (Stockholm, Sweden). In order to ensure an easy and homogenous application of the probiotic strain, a cream was prepared containing the following ingredients: *Helianthus annuus* seed oil (5%), *Prynuns amygdalus dulcis* oil (3%), *Butyrospermum parkii* butter (2%), caprylic/capric triglycerides (5%), stearic acid (2.5%), cetearyl alcohol (4.5%), tocopherol (0.1%), and sterile water (77.9%). Half of the cream (inoculum treatment) was inoculated with 2.2 × 10^5^ CFU.g^−1^, while the other half of the cream was not (control treatment). The cream was prepared each week to ensure the viability of the strain (checked by culture in petri dishes). An evaluation of the microbiological quality was performed in accordance with the ISO 16212 and ISO 21149 standards to control exogenous contamination.

### 2.2. Volunteer Recruitment and Inoculum Application

A preliminary step consisted of the selection of individuals in order to study a homogeneous group of people. Finally, three volunteers (named A, B, and C) were recruited in accordance with the following criteria: being a woman, being Caucasian, being aged between 18 and 25 years old, of normal weight (18.5 < BMI < 24.9), having no skin disease, having no antibiotics for the 6 past months, having healthy diet, not having a tattoo on the arms, and not having a swimming pool either before or during the study. Participation in the experiment required mandatory informed and written consent from the volunteers. The study was conducted during the month of October to avoid UV effects on the skin. The samples were anonymized for researchers before their use, and the study was carried out with the approval of the Ecole de Biologie Industrielle (Cergy, France) ethical committee in accordance with the EU general data protection article (EU Regulation 2016/679). Each morning, following a shower using the same shower gel (neutral pH and without sulfate), 1 mL of each cream was spread on each forearm by the volunteer (one arm for the inoculated treatment and one arm for the control). Each week, 7 aliquots (corresponding to 7 days use) of each type of cream (inoculum and control treatment) were supplied to each volunteer. The cream aliquots were kept at 4 °C until use.

### 2.3. Sampling and DNA Extraction

From the first application (T0) and after one, two, and three weeks of the experiment (T1, T2, and T3, respectively), 3 replicated samples were collected from each forearm, which had been treated or not treated respectively with *L. reuteri* DSM 17938, for each volunteer inside a 23 cm^2^ glass frame with a sterile cotton swab (Classiq Swabs™, Copan Diagnostics, Murrieta, CA, USA), moistened with 120 µL of a sterile solution of NaCl (0.15 mol L^−1^) and Tween 20 (0.1%). Sampling was performed for 30 s using a centrifugal movement by the same technician. To prevent the alteration of microbial diversity [24], each cotton swab head was placed immediately in a microcentrifuge tube and frozen at −20 °C until DNA extraction. The skin microbial DNA was extracted using a NucleoSpin^®^ Tissue (Macherey-Nagel, Düren, Germany). As recommended by the manufacturer, we followed the Dental Swab protocol with the alternative A. Nucleic acids were eluted in a final volume of 30 µL of elution buffer. Concomitantly, 1.10^5^ CFU of *L. reuteri* DSM 17938 was extracted using the same kit to identify the associated genetic marker (see below) and follow the strain colonization dynamics. The absence of microbial contamination was checked by performing an extraction blank.

### 2.4. Molecular Profiling of Bacterial Communities

The skin bacterial community structure was analyzed using the Terminal-Restriction Fragment Length Polymorphism (T-RFLP) profiling method following the protocol of Blaud et al. (2015). Briefly, 16S rRNA genes were amplified using the fluorescently labelled primer FAM-63F and 1389R. A Biorad T100 thermal cycler was used for amplification with the following programs: the initial denaturation was 94 °C for 2 min, followed by 30 cycles of 94 °C for 30 s, 57 °C for 45 s, and 72 °C for 90 s, followed by a final extension time at 72 °C for 10 min. The PCR products were digested using the restriction enzyme *AluI* (ThermoFisher, Wilmington, DE, USA). The samples were analyzed with an ABI 3730 PRISM^®^ capillary DNA sequencer (Applied Biosystems, Foster City, CA, USA). The T-RFLP profiles were analyzed using the GeneMapper^®^ v. 3.7 software (Applied Biosystems, Foster City, CA, USA). Fragments with a relative abundance of a proportion < 0.5% were removed from the matrices because they were too close to the background noise. The 16S terminal-restricted fragment (T-RF) belonging to *L. reuteri* DSM 17938 was removed from the dataset and analyzed separately to provide the colonization dynamic and to ensure that only the indigenous bacterial communities were studied. The richness of the genetic profiles was expressed as the total number of different T-RFs, and the evenness of the profiles was estimated using the Simpson–Yule index: E = 1/∑pi², where pi is the proportion of a given peak [25]. The β-diversity was estimated using relative abundance matrices and multivariate analyses (see below).

### 2.5. Statistical Analysis

All the statistical analyses were performed using R software (1.1.463). After normality (Shapiro) and homoscedasticity (Bartlett) tests, the richness and evenness of the T-RFLP profiles were analyzed with a Three-Way Repeated Measures ANOVA and performed using the rstatix package (0.5.0). A Principal Coordinates Analysis (PCoA) was performed and followed by a Between Class Analysis (BCA) using time and introduction as factors. The differences between the treatments were tested with a Monte Carlo permutation test (999 permutations). In addition, a procrustes analysis was performed using the function “GPA” in the FactoMineR package (2.2) [26]. Histograms and multivariate plots were performed using the ggplot2 package (3.1.1).

## 3. Results

### 3.1. L. reuteri DSM 17938 Skin Colonization Dynamics

**Figure 1:**
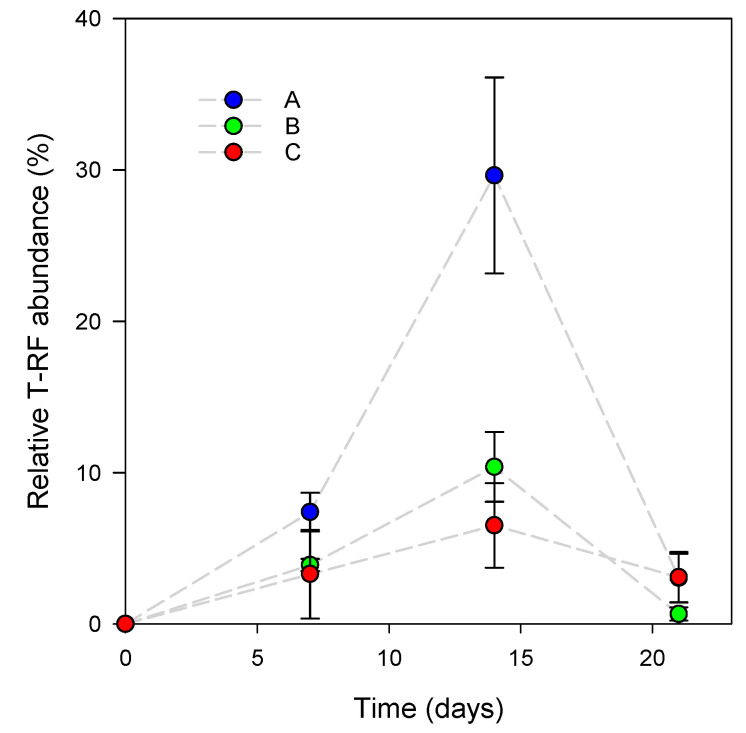
Relative abundance (%) of an *L. reuteri* DSM 17938 restriction fragment during the experiment for each subject (A, B, C). Error bars represent the standard deviation of the mean (*n* = 3).

The colonization of the skin by *L. reuteri* DSM 17938 was monitored by measuring the relative abundance of its associated T-RF (Figure 1). The highest relative abundance of the genetic marker was reached for subjects A and B (30% ± 6% and 10% ± 2%, respectively) after 14 days of the experiment and decreased drastically thereafter to the same level observed after the first 7 days (<10%). For subject C, no significant difference was observed throughout the experiment. The result indicates that the *L. reuteri* DSM 17938 colonization rate was not constant, and that the strain colonization ability decreased throughout the final week of the experiment. The result also indicates that colonization varies between individuals. The overall dynamics of *L. reuteri* DSM 17938-associated T-RFs suggest that the inoculated strain did not maintain the skin colonization rate observed after 2 weeks of treatment, even though the daily application rate did not change until the end of the experiment. The use of a molecular marker does not allow DNA to be discriminated from cells which are dead or alive. Thus, we could not conclude if the strain was able to graft in the microbiota, even at low abundance, or if it failed to establish in the skin habitat. In the first hypothesis, the strain is able to maintain a low-level population, meaning that it gained an ecological niche. Otherwise, the decrease in the T-RF means that the strain disappeared by competitive exclusion due to habitat and/or nutrient unavailability or antimicrobial production by indigenous communities [27]. Using a visualization method such as fluorescent in situ hybridization (FISH) may help in testing these hypotheses [28].

### 3.2. Effect of Probiotics on Skin Bacterial Community Structure

**Figure 2:**
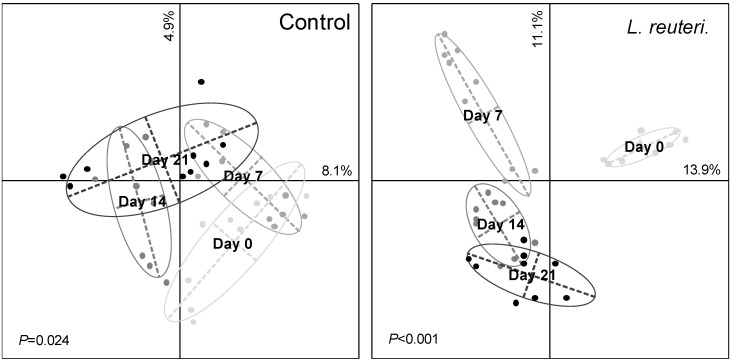
Principal Coordinates Analysis (PCoA) based on the skin bacterial Terminal-Restriction Fragment Length Polymorphism (T-RFLP) profiles during the experiment of control (left) and the introduction of *L. reuteri* DSM 17938 (right). The letter represents the barycenter of the replicates (*n* = 9) for each treatment. The Monte Carlo test-simulated *P* values (lower left corner) revealed significant differences between the sampling dates.

**Figure 3:**
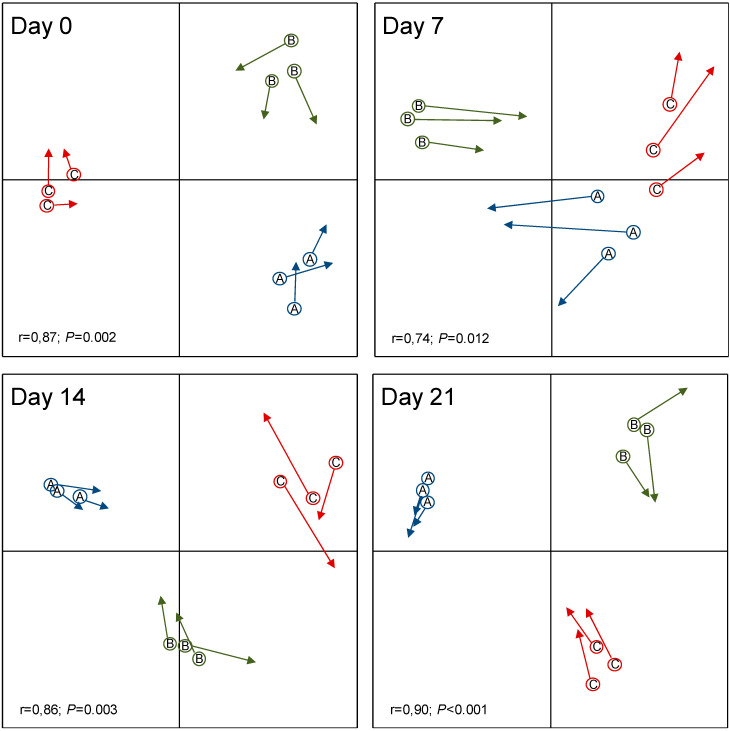
Procrustes analysis using the Bray–Curtis distances between the bacterial communities of the control (circles) and *L. reuteri* DSM 17938-inoculated skin microbiota (arrows) for each subject (A, B, C) after 0, 7, 14, and 21 days of the experiment. The results of the Mantel test (lower left corner) showed a significant correlation between the control and treatment distance matrices.

The number of T-RFs and the evenness of the restriction profiles (Figure S1) did not change significantly during the experiment for either the control or the inoculated treatment (Table 1). However, these two parameters were significantly (*p* < 0.05) higher for the skin microbiota inoculated with *L. reuteri* DSM 17938 than for the control at day 7. The multivariate analysis based on the Bray–Curtis distances and the Monte Carlo permutation test showed that the bacterial community structure changed during the experiment with both treatments (Figure 2), with a higher level of significance (*p* < 0.001) when *L. reuteri* DSM 17938 was inoculated. The first two axes of the PCoA explained 13% and 25% of the variability for the control and the inoculated skin microbiota, respectively. The procrustes analyses evidenced relative changes between the control and the inoculated microbiota distance matrices at each sampling date, (Figure 3). The impact of the *L. reuteri* DSM 17938 inoculum was the greatest after 7 days, when the Mantel test correlation coefficient showed the lowest value (*r* = 0.74). Afterwards, the microbial community structure became more similar, at a same level as at the beginning of the experiment. All the individuals’ skin microbiota were clearly distinguishable, and the most variable in time was that of subject C, which indicates that the effect of the *L. reuteri* DSM 17938 inoculum differs slightly between individuals. Temporal variability in skin microbial communities has been previously observed but remains classified as a minor effect [29]. For the control treatment, it could have been amplified by the excipients applied, as the formula contains lipidic compounds, which can be used by bacteria as carbon and/or an energy source. In skin, oily surfaces could support specific bacterial species that differ from sites. Thus, lipids could change the bacterial relative abundance, as previously observed with lipidic cosmetic products, as bacterial abilities to metabolize components are different depending on the species [30].

In the presence of the *L. reuteri* DSM 17938 inoculum, the bacterial community structure changed. The change in the bacterial community could be the result of a direct or indirect effect. Firstly, the inoculum of *L. reuteri* DSM 17938 could have a direct effect through its tentative implantation, leading to strong competition for ecological niches and the disturbance of the balance of indigenous flora. Secondly, it is possible that the inoculum of *L. reuteri* DSM 17938 has an indirect effect. If the strain fails to establish itself, the products of its lysis could be used as substrates by the indigenous bacterial community, promoting some opportunistic species over others and changing the overall bacterial community structure.

### 3.3. Resistance of Skin Microbiota to Probiotics Colonization

A significant negative relationship was found between the relative abundance of T-RF associated with *L. reuteri* DSM 17938 and the Mantel r correlation between the inoculated and the control matrices (Figure 3). The result suggests that the degree of colonization by the inoculated probiotic is related to the skin bacterial community structure. *L. reuteri* DSM 17938 introduction constitutes a biological disturbance of the skin microbiota. Although this disturbance occurred every day, the bacterial community was not durably affected. The transitory change is the consequence of microbiota resilience. Resilience is defined as the ability of an ecosystem to absorb perturbation and return to its initial state [31,32]. This form of transitory effect following introduction has been observed in various ecosystems, such as soils [33], the human gut [34], and skin, following antibiotherapy [35,36]. In skin, the ecosystem resilience capacity constitutes a hurdle to the implantation of pathogenic microorganisms [37]. A community with a high diversity is more resistant or resilient to invasion because all of the resources and niches have been optimized [34,37,38]. The lack of a highly significant effect by the *L. reuteri* DSM 17938 could be the result of three principal mechanisms. Firstly, it could be due to ecological niches being saturated and thus only being able to host the microorganisms that are already present [39], resulting in unequal competition for niches that favor established strains over invading strains. Secondly, it could be due to competition for nutrients, as skin is an oligotrophic ecosystem and, in a stable environment, all degradation pathways are optimized as cross-feeding [40]. Thirdly, we could hypothesize antimicrobial production by indigenous microorganisms, which is likely to restrain inoculated or pathogen species growth [41,42]. The application of ecological principles in medicine offers a useful framework for a better understanding of the relationship between the human microbiome and human health [12]. The resistance or resilience capacities of indigenous microbial communities [43] have been tested in various environments, but the human microbiome is still documented less. The resilience of the skin microbiota to antibiotherapy has already been studied, but not in relation to exogenous strain introduction. The study has shown for the first time that the repeated introduction of a probiotic (*L. reuteri* DSM 17938) to the skin microbiota only had a small and transient effect on its diversity. The result, obtained in young and healthy people, might differ substantially in cases of dysbiosis, chemical exposure, or disease, when the microbiota is less diverse.

## Figures and Tables

**Table 1:** Summary of the Three-Way Repeated Measures ANOVA (F values), assessing the effects of the *L. reuteri* DSM 17938 introduction (L), sampling time (T), and subject (S) on the richness and evenness.

	Degree of Freedom	T-RF Richness	T-RF Evenness
Lactobacillus (L)	1	66.036 *	171.927 ***
Time (T)	3	9.567 *	21.345 ***
Subject (S)	2	4.136	14.287 **
L × T	3	8.745 *	20.323 ***
L × S	2	4.964	2.873
T × S	6	4.184 *	8.142 *
L × T × S	6	2.089	8.081 *

* 0.05 > *p* > 0.01; ** 0.01 > *p* > 0.001; *** *p* < 0.001.

## Supplementary Materials

The following are available online at https://www.mdpi.com/2076-2607/8/8/1223/s1: **Figure S1**: Number of T-RFs (**a**) and restriction profile evenness (**b**) of the skin bacterial communities without (control) and with application of *L. reuteri* DSM 17938 inoculum during the experiment, for all subjects. Error bars represent the standard deviation of the mean (*n* = 9).
