# Supplementary material for: Human Skin Bacterial Community Response to Probiotic (Lactobacillus reuteri DSM 17938) Introduction
Source: Microorganisms. 2020 Aug 11;8(8):1223. doi: 10.3390/microorganisms8081223 (PMC7465198; doi:10.3390/microorganisms8081223)
Supplement: Supplementary file 1 [file microorganisms-08-01223-s001.pdf]

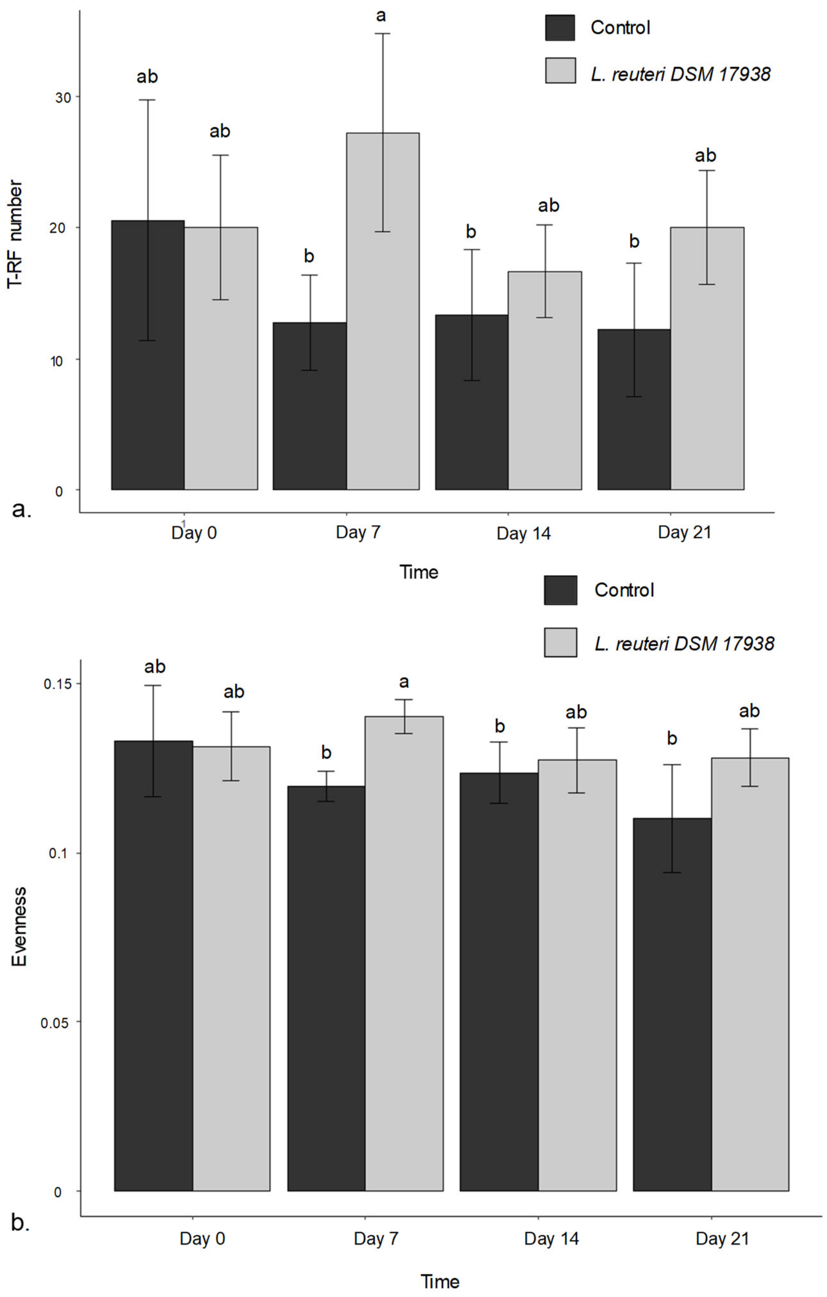

Figure S1. Number of T-RFs (**a**) and restriction profile evenness (**b**) of the skin bacterial communities without (control) and with the application of *L. reuteri* DSM 17938 during the experiment, for all subjects. Error bars represent the standard deviation of the mean ($n = 9$).
